# Severe persistent unremitting dermatitis, chronic diarrhea and hypoalbuminemia in a child; Hartnup disease in setting of celiac disease

**DOI:** 10.1186/s12887-014-0311-6

**Published:** 2014-12-20

**Authors:** Thomas Ciecierega, Imad Dweikat, Mohammad Awar, Maher Shahrour, Bassam Abu Libdeh, Mutaz Sultan

**Affiliations:** New York Presbyterian, Weill Cornell Medical College, New York, USA; An-Najah National University, P.O. BOX 7, Nablus, West Bank Palestine; Makassed Hospital, Alquds University, Medical College, Jerusalem, Palestine

## Abstract

**Background:**

Celiac disease (CD) is a complex autoimmune disorder that can lead to an inflammatory small intestinal villous atrophy and malabsorption.

Hartnup disease is an autosomal recessive disorder caused by increased urinary excretion of neutral amino acids. Co-occurrence of Hartnup disease and CD is extremely rare with only a single case reported.

**Case presentation:**

We report a 3-year girl with chronic diarrhea, Hypoalbuminemia and exfoliative erythema. She was diagnosed with celiac disease, which did not improve on gluten free diet. Hartnup disease was suspected and was confirmed by neutral aminoaciduria. Niacin was started and followed by dramatic improvement.

**Conclusion:**

Presence of Celiac and Hartnup disease in single individual is very rare. Complete nutritional assessment of refractory celiac patient can reveal underlying nutritional deficiency.

## Background

Dermatoses are frequently the presentation of various metabolic and malabsorptive syndromes with Celiac Disease (CD) being the most common one. Celiac disease is an autoimmune, gluten-dependent enteropathy that leads to small bowel mucosal damage causing malabsorption of ingested nutrients. Exfoliative erythema can be found among celiac patients due to malabsorption of many microelements. While Celiac disease is a relatively common disorder in children, Hartnup disease is not.

Co-occurrence of Hartnup disease and CD is extremely rare with only a single case reported.

## Case presentation

**Figure 1:**
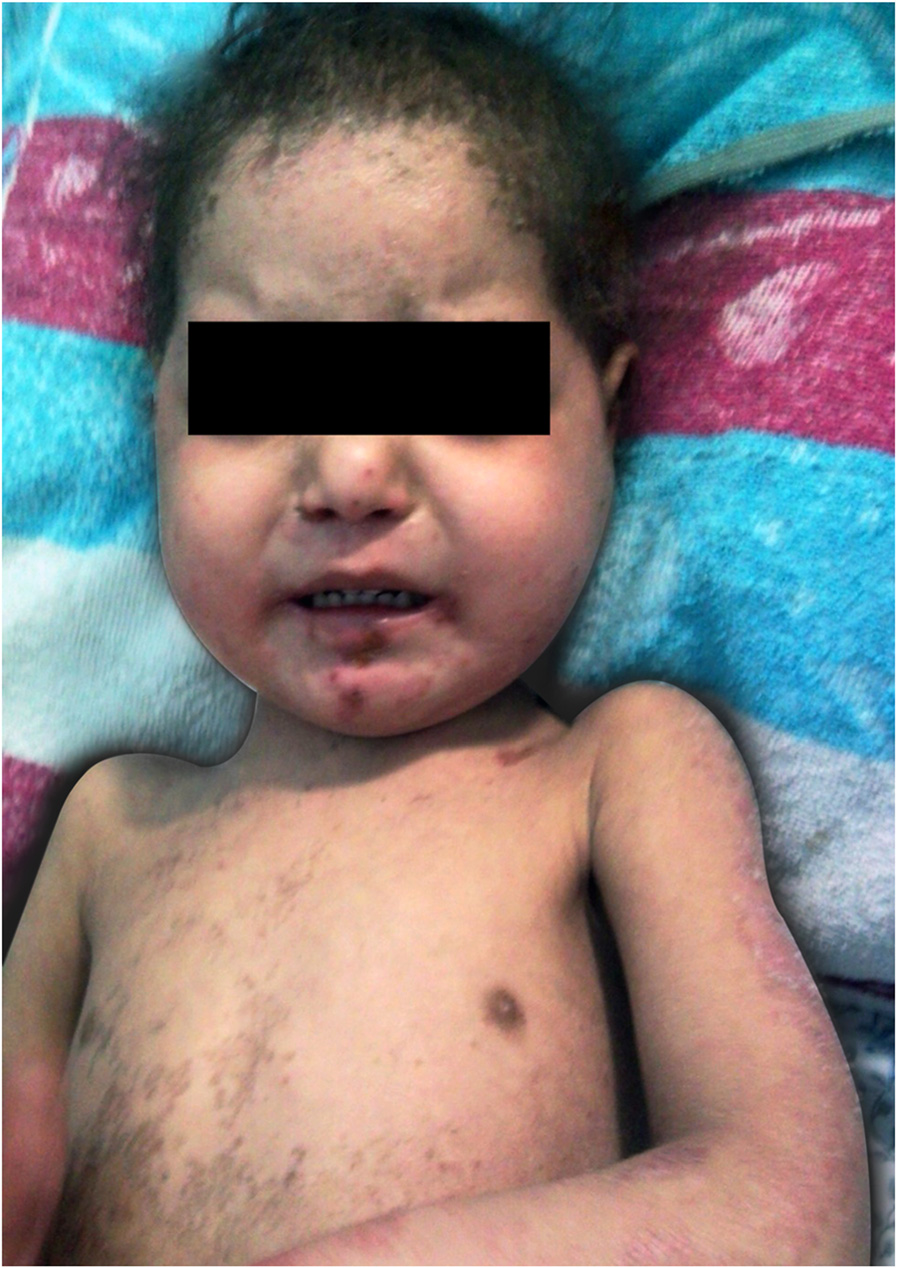
**Initial Presentation: significant photosensitivity with severe scaly desquamating skin rash and hyperpigmentation involving the face, neck, lower and upper extremities.**

A 3-year-old Palestinian girl was referred with primary complains of persistent chronic diarrhea and hypoalbumenemia since one year of age. Additional symptoms included significant photosensitivity and severe scaly skin rash involving face, neck, lower and upper extremities (Figure 1).

On further investigation, she also complained of painful recurring oral ulcers and very low energy level.

She was admitted for severe chronic malnutrition. Her physical exam was significant for abnormally low growth parameters (weight 10 kg and height 83 cm; both were two standard deviations (2SD) below normal), generalized pitting edema and mild abdominal distension without organomegly.

Initial evaluation included labs and stool studies. Abnormalities on initial laboratory evaluation included anemia (Hgb 10.1 g/dl reference range (11.5-16), and coagulation abnormalities (Prothrombin Time17.4 (13-14.5), INR 1.6, (1-1.14). Other pertinent results: alkaline phosphatase 171 U/L (100-320), ALT: 26 U/L (5-45), AST: 34 U/L (10-35), total protein: 6.2 g/dl (6-8), albumin 1.9 g/dl (3.6-5.20). Of note, rheumatoid factor, anti-nuclear antibody, Human Immunodeficiency Virus (HIV) status, vitamin B12, folate, ferritin level and urine organic acids were all normal.

Her Celiac screening was abnormal with tissue transglutaminase IgA antibodies (tTG) >200 U/ml (<12) and total IgA 150 mg/dl (14-123) She underwent upper endoscopy which revealed significant duodenal blunting ad scalloping. Biopsies were consistent with diagnosis of CD (Marsh scale 3b). Patient was started on nasogastric tube feeding with high-protein, gluten-free diet and supplemented with Total Parental Nutrition (TPN), intravenous (IV) zinc sulphate, microelements (Pediatrace) and multivitamins (Infuvite Pediatric). Of note, zinc level returned normal.

**Figure 2:**
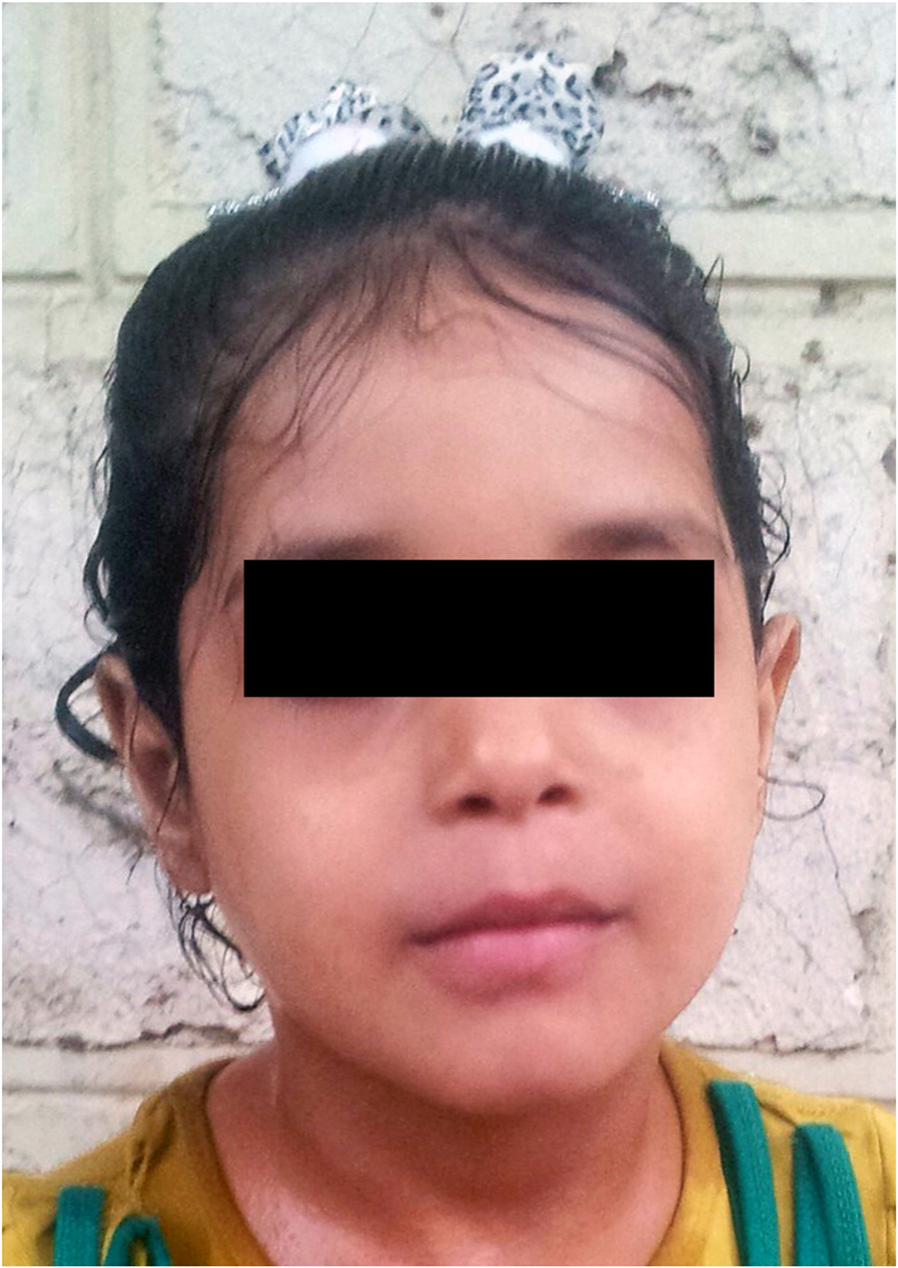
**Patient after treatment: a complete resolution of previous symptoms.**

Patient had only mild clinical and laboratory improvement after 2 weeks of aggressive treatment. She required multiple albumin infusion with only transient improvements. A short-course of parental steroids (Methylprednisolon 2 mg/kg, 10 days) for the suspicion of refractory CD also did not improve her clinical status. Because of combination of non-improving clinical course and persistent symptoms, she was re-evaluated and suspected to have a niacin deficiency (The Pellagra) based on the distribution of the rash, photosensitivity, intractable chronic diarrhea and low mood (the 4D’s: Diarrhea, Dermatitis, Dementia and Death). The patient was started on oral niacin (50 mg three times daily) followed shortly by a dramatic improvement and subsequent complete resolution of her symptoms within four weeks from the diagnosis (Figure 2). Reviewing the dietary history showed that she was consuming decent amount of protein from fish, meat and diary products ruling out primary cause of inadequate intake of niacin. Hartnup disease was considered as a secondary cause of niacin deficiency.

Diagnosis of Hartnup disease was confirmed by presence of neutral aminoaciduria; her urine aminoacids chromatography showed increased levels of excreted neutral amino acids (glutamine, valine, phenylalanine, leucine, asparagine, citrulline, isoleucine, threonine, alanine, serine, histidine, tyrosine, tryptophan). Her urine excretion of proline, hydroxyproline and arginine was normal which differentiates Hartnup disease from other causes of gross aminoacidurea.

She remains on gluten free diet with daily niacin supplementation with no relapse of any of her symptoms.

Although celiac disease is considered a fairly common disease with estimated prevalence of up to 2% in general population (Europe and United States), hartnup disease is an extremely rare disorder. The overall incidence of Hartnup disease by newborn screening program is 1 case per 25,000 births in New South Wales and 1 case per 54,00 in Quebec [1]. There is no available data on the incidence of the disease in Palestine.

CD can be associated with extra-intestinal manifestations. Among them, several skin pathologies have been described [2-4]. Dermatitis herpetiformis is a well-described entity, presenting as an itchy, chronic, papulovesicular eruption, which may leave pigmentation and scarring [3]. Absence of pruritus and vesicular lesions, in addition to non resolving rash after gluten free diet made dermatitis herpetiformis unlikely in our patient.

Exfoliative erythema of malnutrition is a term for skin lesions caused by a combination of multiple deficiencies in vitamins, microelements, essential fatty acids and amino acids. On admission, the provisional diagnosis was malabsorption due to underlying Celiac disease with severe acute malnutrition causing exfoliative erythema. Parenteral nutrition, IV zinc and multivitamin supplementation failed to improve her dermatological manifestations. Dramatic response to niacin supplement was diagnostic for pellagra.

Hartnup disorder is an autosomal recessive abnormality of renal and gastrointestinal neutral amino acid transport [5,6]. The failure to resorb amino acids in this disorder is compensated by a protein-rich diet. However, in combination with a poor diet and other factors like malabsorption, more severe symptoms can develop.

Hartnup disorder was first described in two siblings of the Hartnup family in 1956. Soon after its initial description, it was suggested that Hartnup disease results from a tissue specific failure to resorb neutral amino acids in the kidney and intestine.

The causative gene, *SLC6A19,* is located on a locus on short arm of chromosome 5 (band 5p15.33), which encodes a defective transporter [7]. The transporter is found in kidney and intestine, where it is involved in the reabsorption of all neutral amino acids. An abnormality in tryptophan transport leads to niacin deficiency, which is responsible for the pellagra-like eruptions.

In our case, the severity of the skin lesions can be explained by the coexistence of two diseases. Most likely the combined deficiencies in amino acids and proteins led to exacerbation of hartnup disease, which is asymptomatic in the majority of the cases.

Co-occurrence of Hartnup disease and CD is extremely rare with only a single case reported [8].

## Conclusion

Presence of Celiac and Hartnup disease in single individual is very rare. It appears that CD with its severe enteropathy have uncovered the Hartnup disease and magnified symptoms of niacin deficiency. Complete nutritional assessment of refractory celiac patient can reveal underlying nutritional deficiency.

### Consent

Written informed consent was obtained from the patient’s father her legal guardian, for publication of this Case report and any accompanying images. A copy of the written consent is available for review by the Editor of this journal.

The local ethics committee of makassed hospital for publication approved the case for publication.

## Abbreviation

CD: Celiac disease
